# Effects of Knee Extension Joint Angle on Quadriceps Femoris Muscle Activation and Exerted Torque in Maximal Voluntary Isometric Contraction

**DOI:** 10.3390/biology11101490

**Published:** 2022-10-12

**Authors:** Filip Kukić, Vladimir Mrdaković, Aleksandar Stanković, Duško Ilić

**Affiliations:** 1 Police Sports Education Center, Abu Dhabi Police, Abu Dhabi 253, United Arab Emirates; 2 Faculty of Sport and Physical Education, University of Belgrade, 11030 Belgrade, Serbia

**Keywords:** electromyography, motor control, F–L relationship, RMS–F relationship, relative muscle force

## Abstract

**Simple Summary:**

For human movement to occur, the neural system needs to activate muscles. Muscles then pull the bones that rotate around the joints. As the joint angle for the movement initiation changes, the mechanical conditions for the muscle force production also change, as does the exerted torque (i.e., maximal endpoint force). However, it is unclear whether different joint angles produce different muscle activation in maximal voluntary isometric contraction. This study investigated the effects of knee joint angle on muscle activation, produced torque, and whether the knee angle affects the muscle activation–torque ratio. Participants were instructed to produce maximal isometric knee extension in joint angles of 80, 90, 100, 110, 120, and 130°, while their muscle activation and torque output were assessed. The results confirmed the dependence of torque on knee angle, while muscle activation remained unaffected. While the trend could be observed, significant effects did not occur in muscle activation–torque ratio for lateral and medial quadriceps heads. The investigated range of knee joint angles seems to provide optimal conditions for maximal muscle activation but not for the torque output, hence for the ligament loading and joint contact forces. Therefore, clinicians and sports coaches should carefully set the training goals and choose the knee joint angles accordingly.

**Abstract:**

This study investigated the effects of knee joint angle on muscle activation, exerted torque, and whether the knee angle affects the muscle activation–torque ratio. Nine healthy adult male participants participated in the study. They performed maximal voluntary isometric contraction (MVIC) at six (80°, 90°, 100°, 110°, 120°, and 130°) different knee joint angles (i.e., angles between the thigh and shin bones). Their maximal torque was assessed utilizing an isokinetic chair, while their muscle activation (root mean square [RMS]) was assessed using an eight-channel single differential surface EMG sensor. For the purposes of the torque–knee angle relationship and muscle activation–knee angle relationship, the torque and RMS were normalized relative to the maximal value obtained by each participant. To evaluate the muscle activation–torque ratio in function of knee angle, RMS was normalized relative to the corresponding torque obtained at each knee angle. Repeated measure analysis of variance was used to investigate the effects of knee angle on muscle activation, torque, and muscle activation–torque ratio. There was a significant effect of knee joint angle on normalized torque (F = 27.521, *p* < 0.001), while the activation of vastus lateralis and vastus medialis remained unchanged. The changes in knee angle affected the muscle activation–torque ratio of vastus lateralis (Chi-square = 16.246, *p* = 0.006) but not the vastus medialis. These results suggest that knee joint angles from 80° to 130° provide a stable milieu for muscle electrification, while mechanical factor such as knee joint angle (i.e., lever arm length) affect the torque output when one needs to contract quadriceps maximally during the isometric contraction.

## 1. Introduction

Activation of the motor unit leads to contraction of every muscle fibre innervated by that motor unit which results in the production of a muscle force, whereby force produced by a muscle highly depends on the motor unit activity and mechanically at the actual sarcomere length [1,2,3,4]. Thus, the ability of skeletal muscles to activate and produce forces and the dependence of these forces on muscle length are the most important functional characteristics of skeletal muscles [1,3]. Indeed, human movements are typically angular as muscles are pulling the bones (levers) around the joints (axes) [5], thereby muscle force applied at the bone insertion may produce different endpoint force (i.e., torque) depending on the lengths of moment arm dictated by joint angle [6,7]. Based on these characteristics, athletes may choose sport-specific knee joint angles to initiate effective movements, depending on the desired movement outcome (e.g., maximal strength, power, and speed).

It is a well-established fact that the quadriceps are one of the main skeletal muscles in humans responsible for the mobility, stability and maintenance of the upright position such as walking, running, and kicking [8,9,10,11]. Phylogenesis gifted it with four heads, vastus lateralis (VL), vastus medialis (VM), rectus femoris (RF) and vastus intermedius [12,13]. Although the intensity of activation of each quadriceps head may be affected by the knee joint change [14], they act as one muscle and the force they individually produce is joined and represents a measure of overall quadriceps muscle force [15]. The primary function of quadriceps heads is to extend the knee, while the expression of neural, biological, physiological, anatomical, and mechanical properties of quadriceps depend on the mechanical conditions of the movement that this muscle needs to perform [16,17]. Therefore, choosing the correct knee angle may often have a crucial role in effective movement performance with a lower risk of injury as knee angle affects the force and power outputs [18,19,20], as well as musculoskeletal loading and loading of cruciate ligaments [21,22,23,24]. 

The force produced in multi-joint movements depends on the interplay of intermuscular and intramuscular coordination, while in single-joint movements, muscle force largely depends on intramuscular coordination [1,25]. In a single-joint movement, such as knee extension, only the heads of the quadriceps are activated, and the only adjustment in motor unit recruitment across the range of knee angles (i.e., muscle lengths) occurs between the heads of quadriceps femoris muscle [26]. As the knee angle changes, this may result in different neural and mechanical outputs of quadriceps heads. In that regard, Krishnan et al. [27] found that changes in knee joint angle significantly affected the electromyographic (EMG) activity of VL, RF and VM at the torques of 10–50% of maximal voluntary isometric contraction (MVIC). Similarly, Wathanabe and Akima [28] used 90°, 115°, 140°, and 165° of knee flexion and found significant effects of knee angle on activation of VL, VM, RF and vastus intermedius measured by EMG. Furthermore, Babault et al. [29] reported significant results of muscle length on EMG signal collected from quadriceps in concentric contraction, whereby lower quadriceps activation occurred at short muscle length (145°) compared to intermediate (125°) and lengthened (105°) muscle. Similarly, Saito and Akima [14] found no difference in EMG activity of VL and VM across the knee angles of 90–120°, while the activation at 150° was significantly lower in submaximal contractions. Indeed, the research on longer quadriceps lengths, when the knee angle is smaller than 90°, is scarce, which poses additional questions on the effects of muscle length on quadriceps muscle activation during the MVIC. 

Considering this, the intensity of muscle contraction and the interaction between the muscle length and muscle contraction intensity seem to affect muscle activation. However, whether the muscle activation is affected by changes in muscle length when the requirement is to produce maximal force is not clear. Therefore, the aims of this study were: (1) to investigate the effects of knee joint angle on quadriceps activation (i.e., muscle activation–L relationship); and (2) to investigate the effects of knee joint angle on the ratio between the muscle activation and corresponding torque (i.e., muscle activation–torque ratio). 

Before the investigation of muscle activation, the torque–knee joint angle was confirmed to provide the platform for analysis of muscle activation. Accordingly, we hypothesized that the muscle activation will remain unchanged across the selected knee angles, that the ratio between the muscle activation and the torque output will be affected by changes in knee angle, and that the change in knee angle will affect the torque output produced by quadriceps muscle. The rationale for this study lies in the fact that the muscle activation–knee angle relationship, torque–knee angle, and muscle activation–torque ratio in function of knee angle are the basic functional characteristics of muscles that coaches and clinicians should consider when planning and programming training for their athletes or patients.

## 2. Materials and Methods

### 2.1. Participants

Nine (*n* = 9) healthy male students from the Faculty of Sport and Physical Education voluntarily joined the experiment. Their main characteristics were: age 23 ± 1 years, body mass 80.8 ± 7.8 kg, body height 182 ± 7 cm, and percent of body fat = 11 ± 2%. All participants were involved in strength training a minimum of three times per week for the last three years and were familiar with performing the MVIC. They were instructed to refrain from strenuous physical activity 48 h before the assessment. Their medical history showed no evidence of any knee injury or neurological disorders. All participants were informed about the study design and purpose of the study. They proceeded only after they signed informed consent. The research was carried out in accordance with the conditions of the Declaration of Helsinki, recommendations guiding physicians in biomedical research involving human subjects [30]. The ethical approval was obtained from the ethical review board of the Faculty of Sport and Physical Education, University of Belgrade.

### 2.2. Familiarization and Settings

Participants came to the laboratory on two occasions before the testing day, one week and three days, respectively, to become familiar with the testing procedures, personalize the dynamometer to individual measurements of each participant and mark the electrode positions. The first familiarization session was used for setting the isokinetic chair (Kin-Com, Chattanooga Group, Inc. Chattanooga, TN, USA) according to the participant’s anthropometry and for getting familiar with the contraction mode. The horizontal position of the thigh was fixed, while the back support was adjusted to fix the hip joint position at 90°. The axis of rotation of the dynamometer and the axis of rotation of the knee joint were aligned by moving the head of the dynamometer. The same principle was used to parallelly align the dynamometer’s lever arm with the tibia. Two separate belts were placed across the chest to fix the upper body to the chair, one belt fixed the hips to the chair, and one belt was used to fix the thigh to the chair. The force detection system was positioned 1 cm above the lateral malleolus. The chair positions for every participant and each knee joint angle were marked and written down. The second familiarization session was used to get more familiar with the contraction mode and mark the electrode placement places. The skin was shaved at the designated spots for the electrodes, and a waterproof marker marked the spots so they could be visible on the testing day.

### 2.3. Testing Procedure

The testing procedure was conducted following previously reported procedures that were shown to be reliable [31,32]. All participants took part in a 10-min warm-up protocol consisting of five minutes on a bicycle ergometer and five minutes of dynamic stretching. Following the stretching, the electrodes were fixed in their positions and then participants were positioned in a pre-positioned isokinetic chair. Participants performed three MVICs as quick and strong as possible in six different, randomly selected knee joint angles. The average of three trials was used for the analysis. Tested angles were 80°, 90°, 100°, 110°, 120°, and 130° of knee extension (angle between femur and tibia). The duration of each contraction was 5 s [14,15,29], while the period between two consecutive contractions was 60–120 s. After the testing in one joint angle was finished, participants rested for 3–5 min to fully recover [14,29], and during that time, the next joint angle was set. The maximal torque and muscle activation of VL and VM were recorded for each contraction. Given that the length of the moment arm was not measured and that the contraction was isometric, the torque was represented with endpoint force and expressed in N. Contraction correctness was controlled through the force output, whereby every contraction that contained a stretch-shortening cycle or lasted less than 3 s was repeated until three successful contractions were completed. Subjects could see the force output so they could give their best in every contraction, and the testers verbally encouraged them throughout the contraction. 

### 2.4. Electrode Placement and EMG Signal Acquisition and Processing

The EMG signal from VL and VM of the quadriceps femoris was recorded using an eight-channel single differential surface EMG sensor (Delsys Inc., Boston, MA). Electrodes were placed according to the previously reported procedure [31,33]. In short, the skin was shaved at the location of the electrode placement, cleaned with alcohol, and lubricated with ultrasound gel for better electrical conductivity. The electrodes were placed on the most prominent point of the muscle belly, with a minimum of 3 cm between the electrodes to avoid crosstalk [33,34]. To ensure the electrode position for each joint angle, electrodes were tightly fixed with medical tape over the electrodes and around the leg. Additional tapes were also used to fix the cables to the leg, so the cables could not hang loosely. A ground electrode was placed on the hip joint of the opposite leg as close to the great trochanter as possible. 

A single differential multichannel EMG was used to detect the EMG signal. The signal was amplified by Bagnoli eight-channel system and band-pass filtered with the second-order Butterworth filter (passband ripple 3 dB, attenuation 40 dB, corner frequency 100–400 Hz) following the instruction provided by the manufacturer [35]. The sampling frequency was 2000 Hz, and an A/D converter with 12-bit precision in the voltage range of ±0.002 V. Original Delsys software (Delsys EMG works 4.1, Boston, MA, USA) was used for signal processing. The room temperature was maintained using an A/C set at 22 °C. To estimate muscle activity level, the root mean square (RMS) calculation was used to reflect the power of rectified EMG signal [36]. The RMS was calculated using a moving window of a 100 ms length and 80 ms overlap. For further statistical analysis, the first 2 s of each contraction were normalized relative to the highest MVIC. To synchronize the starting point of signals from the electromyogram and the isokinetic chair, the fourth channel of electromyogram was connected to a voltage sensor positioned at the front edge of the tibia where the tibia was in contact with the lever arm. The voltage change caused by the tibia’s pressure on the lever arm presented the beginning of muscle activation that we used for signal processing.

### 2.5. Normalization

The normalization was conducted according to previously explained procedures [29,37]. In short, the MVIC torque at each angle was normalized with respect to the maximal MVIC torque observed across all joint angles. The MVIC EMG amplitude at each angle was normalized with respect to the maximal MVIC EMG observed across all joint angles of each subject. In addition, for the muscle activation–torque relationship, the EMG amplitude at each joint angle was normalized with respect to the corresponding exerted torque value.

### 2.6. Variables

Six variables were defined for further analysis:

1. Torque—Normalized torque from each MVIC with respect to the highest torque obtained by each participant, expressed in %;
2. VL_RMS_—Normalized RMS of VL with respect to maximal RMS, expressed in %;
3. VM_RMS_—Normalized RMS of VM with respect to maximal RMS, expressed in %;
4. ΔVL_RMS_—RMS of VL relative to exerted torque in the corresponding knee angle, expressed in %;
5. ΔVM_RMS_—RMS of VM relative to exerted torque in the corresponding knee angle, expressed in %.

### 2.7. Statistical Procedures

All statistical procedures were conducted in Statistical Package for Social Sciences (IBM, SPSS Statistics 20, Chicago, IL, USA) and R stats (version 4.1.0, 2021). Variables were analyzed descriptively for each tested angle’s mean and standard deviation (SD). The Shapiro–Wilk test was used to check for the normality of distribution. Linear and polynomial regression analysis was initially performed to determine the association of knee joint angle with RMS, torque, and muscle activation–torque ratio (see Supplementary Table S1). The regression analyses showed a significant association of knee angle with torque (R^2^ cubic = 0.697, *p* < 0.001), while VL_RMS_, VM_RMS_, ΔVL_RMS_, and ΔVM_RMS_ were not associated with the knee angle. A multivariate analysis of variance (MANOVA) was further used to test the effects of knee angle on torque and muscle activation. Where the sphericity was violated, a Greenhouse-Geisser correction was used. The pairwise comparison of Fmax and VM_RMS_ between the knee angles was analyzed by the Bonferroni post-hoc adjustment. The normality of distribution for VL_RMS_ and ΔVL_RMS_ at 80° and 120°, and for ΔVM_RMS_ at 80° and 120° was violated (Shapiro–Wilk *p* < 0.05), so the Friedman test and Conover pairwise test were performed. The significance level was set at *p* < 0.05. Cohen’s effect sizes (*d*) were calculated and interpreted as small (*d* = 0.2–0.5), medium (*d* = 0.5–0.8), large (*d* = 0.8–1.3), and very large (*d* > 1.3) [38]. Due to the relatively small sample size, we used a critical F of 2.485, and an effect size of *d* = 1.6 for *p* < 0.05 to indicate significance as calculated by G*Power.

## 3. Results

The descriptive statistics for the mean and standard deviation (SD) of each investigated variable at each tested knee angle are shown in Table 1. The MANOVA revealed that the knee angle affected the torque and ΔVL_RMS_.

**Figure 1:**
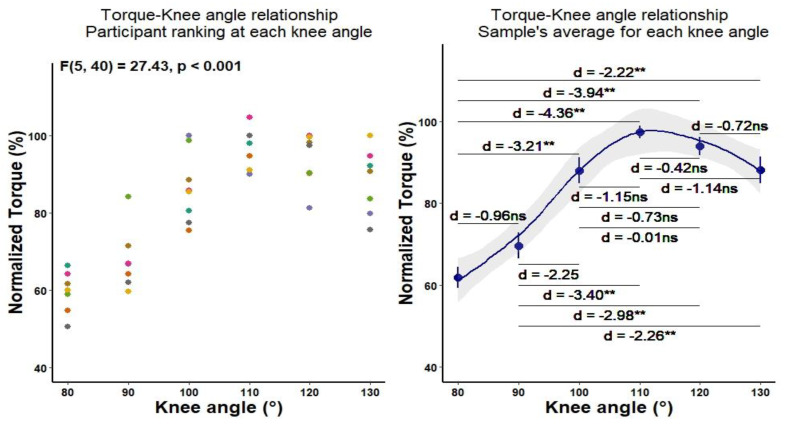
The effects of knee angle change on torque exertion. ** Significant at *p* < 0.01.

The Bonferroni post-hoc analysis revealed a significant effect of knee angle on torque, whereby torque was increasing as the knee extended (i.e., quadriceps muscle was shortening) until the 110° (See Table 2 and Figure 1). As the knee was extending from 110–130° the torque was gradually decreasing (i.e., 110° > 120° > 130°). Although the differences between these knee angles were not statistically significant, the effect size analysis detected small to large differences, suggesting that the effects of knee angle on torque may still exist.

**Figure 2:**
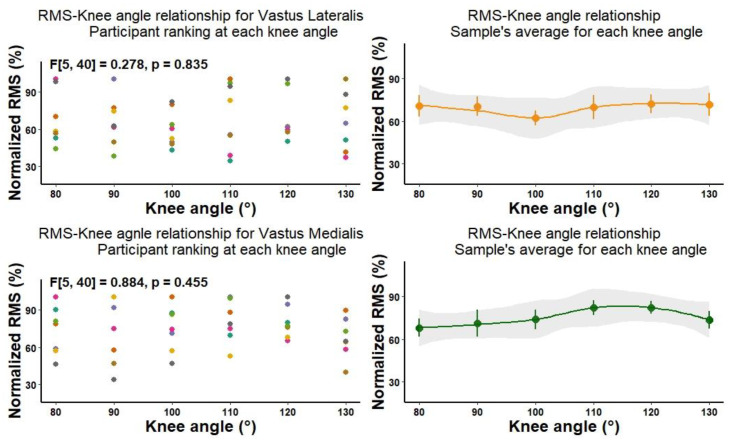
The effects of knee angle on muscle activation of vastus lateralis and vastus medialis.

Normalized EMG of VL and VM were not affected by the changes in knee angle (Figure 2). The individual variations in muscle activation at different knee joint angles and participant ranking indicate the ability of participants to similarly activate lateral and medial quadriceps heads across the selected knee angles.

**Figure 3:**
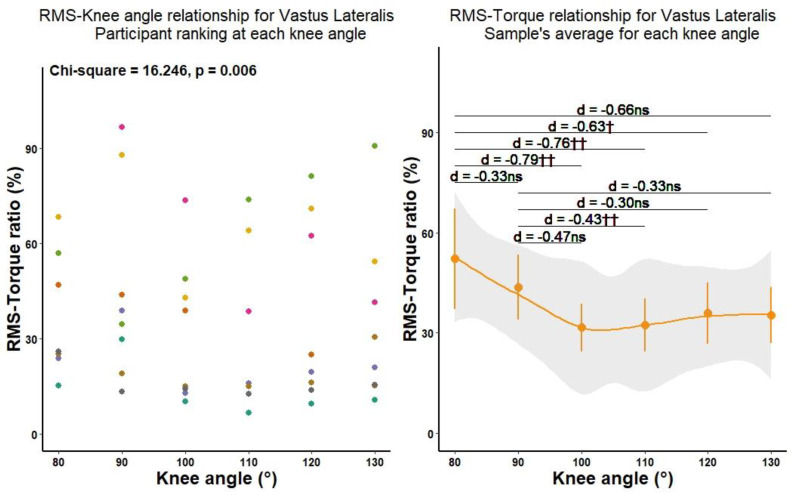
The vastus lateralis RMS—F ration at different knee joint angles. † Significant by Friedman test at *p* < 0.05, †† Significant by Friedman test at *p* < 0.01.

The muscle activation–torque relationship was not affected by knee angle in ΔVM_RMS_ (F[5] = 1.515, *p* = 0.251), while the non-parametric Friedman test of differences showed a significant effect of knee angle on the muscle activation–torque relationship in ΔVL_RMS_ (Figure 3). Pairwise comparisons showed significant differences in muscle activation–torque relationship of ΔVL_RMS_ in relation to knee angle. As the knee extended from 80° towards more extended knee angles (i.e., muscle shortening), the proportion of activation in produced torque gradually reduced (Table 3). A significantly lower proportion of activation was found at knee angles of 100°, 110°, and 120° compared to 80° as well as at 110° compared to 90°. It is of note, however, that the effect sizes were below the level of 1.6, which G*Power determined as the level of significance. Therefore, these results could be interpreted as trends rather than the significant effects of muscle length on muscle activation–torque ratio.

## 4. Discussion

This study aimed to analyze the effects of knee joint angle on muscle activation of quadriceps femoris and investigate the effects of knee joint angle on the ratio between muscle activation and torque. Our results confirmed that the changes in knee angle significantly affect torque exertion. The main findings of this study suggest that normalized RMS of VL and VM remained unaffected. Thereby the first hypothesis was true. The ΔVM_RMS_ (i.e., RMS of VM–torque ratio) remained unaffected by changes in quadriceps length, while the trend in changes in ΔVL_RMS_ (i.e., RMS of VL–torque ratio) could be observed at different joint angles. Given that this trend could not be statistically significant, the second hypothesis was false.

A gradual increase in torque occurred as the knee extended (i.e., the quadriceps were shorter) until the angle of 110°, after which the torque remained unchanged. The torque–joint angle relationship has been well documented in the literature [1,5], wherein changes in joint angle dictate the length of the moment arm of external force, thus affecting the exerted torque. In addition, studies found that muscle forces were lower on shorter and longer muscle lengths, while they remained relatively stable around the middle of the muscle length [3,28,37,39]. This is due to the anatomical and physiological properties of the muscle sarcomere, as muscle force production depends on cross-bridge overlap, and the optimal overlap allows for the highest levels of active tension [1,39]. To that end, mechanical and physiological determinants of torque exertion are affected by changes in joint angle.

For clinical practice, it is worth noting that variations in torque production, as well as manipulations with muscle lever arm and angle of muscle attachment to the bone via changes in knee joint angle, could be used to produce targeted joint contact forces and musculoskeletal loading [40,41,42]. Understanding these mechanisms is also very important for sports practice because for the best physical performance, athletes are typically required to perform in knee joint ranges that provide them with the best potential to utilize muscular activities [19,43,44]. This may lead to an overload of muscles, tendons, ligaments or joints, resulting in tissue injury [23,24]. Therefore, the knee joint angles should be thoroughly analysed when implementing the training program in clinical and high-performance settings.

The results suggest that for the selected range of motion, VL and VM muscle activation was not affected by changes in knee angle. The activation pattern did not have a systematic character. Participants instead showed different individual patterns across the tested range of motion, whereby the variations were relatively small (0.2–14.2%) in both tested muscles. Therefore, participants could activate muscles maximally at each tested knee angle. Our results are similar to those reported by Saito and Akima [14], whose participants showed stable maximal activation of VL and VM at knee joint angles of 90° and 120°.

Furthermore, Wathanabe and Akima showed similar results for the normalized RMS of VL and VM at 90° and 115° of knee joint angle. However, both studies showed a significant decrease in muscle activation at longer muscle lengths (e.g., 145° and longer). This could be explained by similar pennation angles of muscle fibres at muscle lengths belonging to knee joint angles of 80°–130° as the muscle shortening of this range was not sufficient to cause significant changes in pennation angle [45], thus on EMG signal [46]. In contrast, smaller and larger joint angles may activate inhibitory mechanisms related to the function of muscle spindles and Golgi tendon organs that are sensitive to muscle length and muscle tension [47,48,49]. While our results indicate a good positioning and fixation of electrodes, a wider range of knee joint angles could be used in the future, followed by additional analyses of muscle tissue properties and function (e.g., ultrasound, MRI imaging, twitch interpolation, twitch contraction).

Considering that the knee angle did not significantly affect the muscle activation while the torque output increased, the observed trend of changes in the proportion of VL activation in attained muscle force seems logical. However, this further supports the aforementioned arguments that the selected knee angles did not provide sufficient muscle length change to produce significant muscle activation changes. On the other hand, the selected range of knee angles was sufficient to show the changes in torque output, which could be attributed to changes in mechanical conditions of the knee extension movement that change with the knee angle (i.e., an increase of moment arm). Furthermore, physiological advantages could also contribute to an increment in muscle force as the cross-bridge overlap is optimal at about the middle of muscle length [50,51], which in the case of knee extension is between 100 and 120°. To that end, our results provide a good illustration of the neuromechanical components of movement. Our results suggest that in the selected knee angles, VL and VM can generate maximal activation without being inhibited, thereby allowing biological, physiological, and biomechanical factors to be maximally expressed.

### Limitations

A few limitations of this study should be pointed out. Regarding the study sample, it could have been larger, it could include female participants, it could consist of a wider population with varying physical fitness levels, and it could include a wider age span. These issues should be addressed in future studies. The knee angle range could consist of smaller and larger joint angles, which should also be addressed in future studies. Future studies should focus on clinical populations such as pre- and post-operative patients, patients with sarcopenia, and patients with impaired muscle strength.

## 5. Conclusions

This manuscript investigated the effects of knee joint angle on muscle activity of superficial quadriceps heads, exerted torque, and muscle activation–torque interplay during MVIC. The results showed that, for investigated range of joint angles, although exerted torque changes with knee joint angle, the muscle activation remains stable. Considering this, it could be concluded that knee joint angles from 80 to 130° provide a stable milieu for muscle electrification, while mechanical factors affect the torque output when one needs to contract the quadriceps maximally during the isometric contraction. For clinical practice, our results suggest that the treatments for muscle activation of medial and lateral heads of the quadriceps could include the investigated range of angles. However, further analysis and diagnosis are needed to determine angles that produce safe ligament loading and joint contact forces. For sports practice, the results suggest that athletes may be able to produce high levels of quadriceps activation in all investigated knee joint angles, but the torque outputs will change as a function of knee joint angle. Therefore, determining optimal joint angles is crucial for athletic performance.

## Figures and Tables

**Table 1:** Descriptive statistics.

Variable	80°	90°	100°	110°	120°	130°
	Mean ± SD	Mean ± SD	Mean ± SD	Mean ± SD	Mean ± SD	Mean ± SD
Torque (%) **	61.51 ± 7.70	69.30 ± 9.82	87.57 ± 9.81	96.88 ± 3.99	93.46 ± 6.30	87.63 ± 9.34
VL_RMS_ (%)	70.65 ± 22.49	70.44 ± 20.68	62.14 ± 15.70	69.80 ± 25.29	72.11 ±20.43	71.52 ± 24.11
VM_RMS_ (%)	68.06 ± 19.61	71.04 ± 28.24	73.94 ± 20.30	82.08 ± 15.56	82.27 ± 12.95	73.48 ± 18.82
ΔVL_RMS_ (%) †	52.14 ± 45.21	43.53 ± 29.21	31.54 ± 21.32	32.23 ± 23.65	35.83 ± 27.54	35.22 ± 25.29
ΔVM_RMS_ (%)	38.21 ± 31.91	34.87 ± 29.58	27.69 ± 17.39	26.38 ± 13.17	27.15 ± 11.83	26.19 ± 13.80

† Significant at *p* < 0.05 by Friedman test, ** Significant at *p* < 0.001.

**Table 2:** Bonferroni post-hoc analysis for torque.

Pairwise Comparison		Mean Difference	95% CI for Mean Difference	
			Lower	Upper
80°	90°	−7.79%	−19.7	4.1
	100°	−26.05%	−37.9	−14.2
	110°	−35.37%	−47.3	−23.5
	120°	−31.95%	−43.8	−20.1
	130°	−26.12%	−38.0	−14.2
90°	100°	−18.26%	−30.1	−6.4
	110°	−27.58%	−39.5	−15.7
	120°	−24.17%	−36.1	−12.3
	130°	−18.33%	−30.2	−6.5
100°	110°	−9.32%	−21.2	2.6
	120°	−5.90%	−17.8	6.0
	130°	−0.07%	−11.9	11.8
110°	120°	3.41%	−8.5	15.3
	130°	9.25%	−2.6	21.1
120°	130°	5.83%	−6.1	17.7

**Table 3:** Conover post hoc test for ΔVL_RMS_.

Pairwise Comparison		Mean Difference	95% CI for Mean Difference	
			Lower	Upper
80°	90°	8.44%	−18.57	35.46
	100°	20.56%	−6.46	47.57
	110°	19.67%	−7.35	46.68
	120°	16.22%	−10.80	43.24
	130°	17.00%	−10.02	44.02
90°	100°	12.11%	−14.91	39.13
	110°	11.22%	−15.80	38.24
	120°	7.78%	−19.24	34.80
	130°	8.56%	−18.46	35.57
100°	110°	-0.89%	−27.91	26.13
	120°	−4.33%	−31.35	22.68
	130°	−3.56%	−30.57	23.46
110°	120°	−3.44%	−30.46	23.57
	130°	−2.67%	−29.68	24.35
120°	130°	0.78%	−26.24	27.80

## Data Availability

Data is available upon request at filip.kukic@gmail.com.

## Supplementary Materials

The following supporting information can be downloaded at: https://www.mdpi.com/article/10.3390/biology11101490/s1, Supplementary Table S1.
