# Supplementary material for: Effects of Knee Extension Joint Angle on Quadriceps Femoris Muscle Activation and Exerted Torque in Maximal Voluntary Isometric Contraction
Source: Biology (Basel). 2022 Oct 12;11(10):1490. doi: 10.3390/biology11101490 (PMC9598811; doi:10.3390/biology11101490)
Supplement: Supplementary file 1 [file biology-11-01490-s001.zip › biology-1909439-supplementary.pdf]

Supplementary Table S1. Regression analyses for Torque, RMS, and RMS–Torque ratio.

Dependent Variable: Torque

Equation	Model Summary				
	R Square	F	df1	df2	Sig.
Linear	.479	47.891	1	52	.000
Quadratic	.692	57.212	2	51	.000
Cubic	.697	58.740	2	51	.000
Power	.542	61.457	1	52	.000
Exponential	.498	51.611	1	52	.000

Dependent Variable: VLRMS

Equation	Model Summary				
	R Square	F	df1	df2	Sig.
Linear	.002	.084	1	52	.773
Quadratic	.009	.237	2	51	.790
Cubic	.009	.226	2	51	.798
Power	.000	.019	1	52	.890
Exponential	.001	.033	1	52	.856

Dependent Variable: VMRMS

Equation	Model Summary				
	R Square	F	df1	df2	Sig.
Linear	.030	1.605	1	52	.211
Quadratic	.055	1.498	2	51	.233
Cubic	.057	1.548	2	51	.223
Power	.043	2.335	1	52	.133
Exponential	.039	2.129	1	52	.151

Dependent Variable ΔVL_{RMS}

Equation	Model Summary				
	R Square	F	df1	df2	Sig.
Linear	.032	1.746	1	52	.192
Quadratic	.057	1.548	2	51	.222
Cubic	.057	1.548	2	51	.222
Power	.038	2.030	1	52	.160
Exponential	.033	1.801	1	52	.185

Dependent Variable: ΔVM_{RMS}

Equation	Model Summary				
	R Square	F	df1	df2	Sig.
Linear	.040	2.189	1	52	.145
Quadratic	.049	1.326	2	51	.275
Cubic	.049	1.326	2	51	.275
Power	.021	1.105	1	52	.298
Exponential	.019	1.017	1	52	.318
